# Medicinal plants in an urban environment: the medicinal flora of Banares Hindu University, Varanasi, Uttar Pradesh

**DOI:** 10.1186/1746-4269-3-35

**Published:** 2007-11-08

**Authors:** Archana K Verma, Munesh Kumar, Rainer W Bussmann

**Affiliations:** 1 National Academic Counselor, Environment, IGNOU Centre, Mizoram Aizawl, India; 2 Department of Forestry, HNB, Garhwal University, Srinagar Garhwal, Uttarakhand, India; 3 Department of Geography and the Environment, The University of Texas at Austin, 1 University Station A3100, Austin, TX 78712-1098, USA

## Abstract

Varanasi is one of the oldest continuously inhabited cities of the world, and one of the most important Hindu pilgrimage sites. Despite this importance, very little information exits on the cities flora in general, and medicinal species found within its limit in particular. Traditional medicine plays a large role in Indian society. The presented study attempted to investigate if traditional plant use and availability of important common medicinal plants are maintained in urban environments. The paper presents information on the traditional uses of seventy-two plant species collected form the campus of Banares Hindu University, Varanasi, Uttar Pradesh, and highlights the uses of these plants by the local inhabitants.

## Background

Traditional medicine based on herbal remedies has always played a key role in the health systems of many countries. In India the native people are exploiting a variety of herbals for effective curing of various ailments. The plant parts used, preparation, and administration of drugs vary from one place to other. However, the knowledge of herbal medicines is gradually perishing, although some of the traditional herbal men are still practicing the art of herbal healing effectively. These plants are frequently used by the local inhabitants of the area for treatment of various diseases. Ethno-medicinal studies have offered immense scope and opportunities for the development of new drugs. Some modern drugs have been deducted from folklore and traditional medicines. Living close to nature, traditional societies have acquired unique knowledge about the use of wild flora and fauna, most of which are unknown to the people who live away from such natural ecosystem as forests. After years of observations and analysis, trials, error, experimentation or even use of intuitive methods the innovative member of human communities have selected/identified useful and harmful members of the flora and fauna.

Such knowledge and practices/experience were subjected to further modification or enriched with new knowledge of practice by succeeding generations and become a part of the tradition, culture, art, belief, folklore and knowledge base of these traditional communities. The traditional knowledge, skill and practices thus developed are freely exchanged cared for and nourished as a common property of the communities [1].

The value and importance of traditional knowledge are now being increasingly acknowledge all over the world. The pharmaceutical industry continues to investigate and confirm the efficacy of many medicines and toxins used by traditional communities [2].

The forests have been the source of invaluable medicinal plants since the time man realized the preventive and curative properties of plants and started using them for human heath care. The old traditional Indian Systems of Medicine (ISM), is one of the most ancient medicine practices known to the world, and derives maximum formulations from plants and plant extracts that exist in the forests. About 400 plants are used in regular production of Ayurvedic, Unani, Siddha and tribal medicine. About 75% are from tropical and 25% from temperate forests. 30% of preparations are derived from roots, 14% bark, 16% whole plants, 5% flowers, 10% fruits, 6% leaves, 7% seeds, 3% wood, 4% rhizomes 6% stems and only less than 20% of the species used are cultivated [3].

Forest degradation processes adversely affect the resource base of medicinal plants. The rural poor, whose dependence on these products is very heavy, are the worst sufferers. The problems are compounded by market-demand driven harvesting without any concern for representation and conservation. In the process essential regenerative components of a plant like roots, tubers, fruits, seeds flowers and bark are indiscriminately collected, leading to degradation and depletion and even extinction of particular species. Due to this ruthless exploitation, many important medicinal plants species are becoming rare and some of them are critically endangered. It is estimated that 10% of all plant species are currently endangered in India [4].

The study was performed in and around the area of Banaras Hindu University campus. Varanasi is one of the oldest continuously inhabited cities of the world, and one of the most important Hindu pilgrimage sites. Despite this importance, very little information exits on the cities flora in general, and medicinal species found within its limit in particular.

The city lies at latitudes 82°50' E to 83°03' E and longitude 25°10' N to 25°25' N at an altitude of approximately 79.1 m above the sea level, with fairly level topography. Varanasi has a humid subtropical climate with high variation between summer and winter temperatures. Summers are long, from early April till October, with the monsoon season in between. Cold waves from the Himalayan region dip temperatures across the city in the winter from December to February. The average temperature is 32°C-46°C in summer; 5°C-15°C in the winter. The average annual rainfall is 1110 mm. Fog is common in winter while hot dry winds called loo blow in summer. Soils are generally old alluvial deposits of the middle Gangetic plain [5].

## Methods

A plant inventory was conducted in and around the campus of Banaras Hindu University [6]. All plants were collected, identified, and vouchers were stored at the herbarium of the Department of Dravya Guna, Faculty of Ayurved, Institute of Medical Science, Banares Hindu University, under the first author's collector series.

The field survey covered different seasons. The survey was started in rainy seasons (August) and collections were repeated every month for two years. Seasonal variations and frequency of plant occurrence were noted.

Ethnomedicinal uses of the plants were first extracted from the relevant literature available in the library of the university [7-10]. The ethnomedicinal uses mentioned in literature were then cross checked through interviews with local inhabitants in the villages and urban areas surrounding the university campus and visits to the local Kavirag and Vaidyas who act as are plant collectors and local healers. The interviews were conducted randomly after obtaining prior informed consent of the participants. Only those ethnomedicinal uses agreed upon by a majority of informants were retained. The study did not involve interviews with tribal ethnic groups, and the dosages, specific formulations and mode of administration were recorded, but retained as intellectual property of the informants.

During the field visit the survey of data collection was made in different places i.e. waste lands, bare lands, play ground, road side, agricultural farms and near other localities. The collected samples of plants were brought to the department for identification.

Data on medicinal/ethnomedicinal uses of plants are presented in additional file 1 in the following sequences: serial number, botanical name, family, vernacular name, part used, life form and ethnomedicinal uses.

## Results and Discussion

India has a tradition of codified healthcare systems: Ayurveda, Unani and Siddha, functions mainly through (1) folk stream and (2) classical stream. The former, is based on oral traditions, practiced by villagers and the tribal communities while the later comprises the codified systems supported by theoretical knowledge, experimental and philosophical explanations provided by many learned physicians and surgeons of earlier time like Charak, Sushruta, Galen, Rhazes, Avicenna, and others. The preventive, corrective and curative approaches of health is the basic strength of the Indian Systems of Medicine (ISM), which are mostly plant based and comprise over 8000 medicinal and aromatic plants species. In India, about 1.5 million practitioners of ISM use around 25000 effective plant based formulations. According to the all Indian co-ordinate project sponsored by the Ministry of Environment of Forests, New Delhi, 40% of the 16,000 recorded flowering plants in India have ethno-medicinal value, whereas, only 10% of these are used in drug and pharmaceutical industries. The intrinsic importance of these medicinal plants can very well prove as a potential source of new drugs [11].

No survey of naturally growing medicinal plants of the present study area has been reported till date. Duthie's work on the flora of upper Gangetic plain [12] and of the adjacent Siwalik and Sub-Himalayan tracts included only few species from the Varanasi area. Mishra [13-16] provided short descriptions of the vegetation of Raj Ghat Ravines, Banaras Hindu University, campus and low laying lands and reported 115, 137 and 44 plant species respectively.

The study found that the plants recorded (Additional file 1) from the site are highly valuable for medicinal uses including diarrhea, dysentery, gonorrhea, leprosy, paralysis, piles, purgative, stomach complaints, ulcer, arthritis, wounds, cholera, diabetes liver complaints, skin diseases, syphilis, throat infections urine complaints, snake bite, body swelling, tumor, malaria, menstrual complaints, rheumatic, skin diseases, swelling, tonic, pulmonary tuberculosis, dog bite, eye diseases, hyperactivity, hydrophobia and lumbago. The study provides sufficient ground to believe that the traditional medicinal practice using native medicinal plants is alive well functioning in the study area. Many communities use wild plant parts for the primary healthcare, due to belief in its effectiveness, lack of modern medicines and medication and poor economic status of people. The treatment of disease with plants and plant products also causes little site effects and is cost effective too.

Keeping the importance of plants in consideration, large numbers of commercially important medicinal plant species are over-exploited by persons involved in the trade. Lack of sustainable harvesting methods, inadequate knowledge about forest management and lack of financial resources are the main causes of over-exploitation, indiscriminate collection and over-exploitation of some commercially important species, and the populations of valuable species are decline.

Man-made extinction of species and habitat degradation are the order of the day. This bio-depletion is due to the exponential geometric growth of human population and man's technological capacity to exploit natural resources in an unsustainable way [17]. Intensive and unrestricted grazing, year after year by multitudes of cattle and goats of local villager, accompanied with spread pilferage from the wild has led to serious decline of medicinal plants in particular and the entire natural environment as whole. More than 95% of the medicinal plant material used by the medicinal plant industry is acquired from the wild, more often than not by illegal means [18].

## Conclusion

The area of Varanasi has been settled for millennia, and represents now a large urban environment. Despite dense urbanization, medicinal plants still play a key role in the health care of the local population. Plants commonly used as traditional medicines in rural areas could still be found in the city, and were collected and used by the local population. Local Kavirag and village Vaidyas often collected the medicinal plants from the area of Benares Hindu University campus when they enter for grazing their cattle. The current over-exploitation and soil compaction due to trampling seems to limit the ability of some species to propagate. Therefore, there is immediate need to conserve these important species for sustainable uses for the future. Efforts should be taken to start sustainable cultivation and harvesting programs in the city of Varanasi.

## Supplementary Material

Additional file 1 Ethnomedicinal uses of medicinal plants in Varanasi. Scientific and vernacular names, life forms and uses of medicinal plants of Banares Hindu University, Varanasi. Click here for file
