# Supplementary material for: Medicinal plants in an urban environment: the medicinal flora of Banares Hindu University, Varanasi, Uttar Pradesh
Source: J Ethnobiol Ethnomed. 2007 Nov 8;3:35. doi: 10.1186/1746-4269-3-35 (PMC2186320; doi:10.1186/1746-4269-3-35)
Supplement: Additional file 1 — Ethnomedicinal uses of medicinal plants in Varanasi. Scientific and vernacular names, life forms and uses of medicinal plants of Banares Hindu University, Varanasi. [file 1746-4269-3-35-S1.pdf]

**Additional file 1- Ethnomedicinal uses of medicinal plants in Varanasi**

| *Sl #* | *Scientific Name* | *Family* | *Part used* | *Life form* | *Ethnomedicinal uses* |
|---|---|---|---|---|---|
| 1 | *Asteracantha longifolia* Nees. | Acanthaceae | Whole plant | H | Aphrodisiac, body pain, diuretic, dropsy, impotency, jaundice, malaria, rheumatism, face & body swelling, tuberculosis and fistula |
| 2 | *Barleria prionitis* L. | Acanthaceae | Root, leaves | S | Anasarca, boils, cough, dropsy, ear complaints, gum trouble, laceration, cracking of feet, to make feet tolerant to hear, piles, rheumatism and tooth ache |
| 3 | *Trianthema portulacastrum* L. | Aizoaceae | Whole plant | H | Jaundice, kidney, and liver complaints |
| 4 | *Achyranthes aspera* L. | Amaranthaceae | Different parts includin g seeds | H | Anti-fertility in women, asthma, bleeding in delivery, boil, bronchitis, child birth , cold, cough, disability, dropsy, dog bite, dysentery, ear complaints, head ache, hydrophobia, insect bite, leucoderma, liver complaints, pain in ribs, body, piles, pneumonia, renal complaints, pheum, scabies, scorpion bite, skin diseases, snake bite, sore, stomach ache, syphilis, tooth ache, whooping cough, wounds and eye complaints |
| 5 | *Aerva lanata* (L.) Schult. | Amaranthaceae | Whole plant | H | Anti-helmintic, boils, cuts, wounds, burns, cholera, diarrhea, dropsy, ear complaints, fever, head ache, malaria, skin disease, snake bite, and swelling and white discharge in urine |
| 6 | *Alternanthera sessilis* (L.) R.Br. ex DC. | Amaranthaceae | Leaves, stem | H | Bone-fracture, eye complaints, hydrophobia, diarrhea, dysentery, malarial fever and night blindness |
| 7 | *Centella asiatica* (L.) Urb. | Apiaceaeae | Whole plant | CH | Anti-helmintic, blood disease, blood dysentery, brain tonic, cholera, cooling, cough, diarrhea, diuretic, dysentery, eczema, eye complaints, fever, gastric disorder, head ache, leprosy, liver complaints, memory, nervous disorder, tonic, respiratory disorder, skin diseases, stomach ache, syphilis, tuberculosis, tumor, boil, urine complaints and wounds |
| 8 | *Alstonia scholaris* (L.) R.Br. | Apocynaceae | Leaves, bark, latex, flower | T | Asthma, bronchitis, chest pain, child birth, cholera, diarrhea, eye complaints, fever, malaria, pneumonia, rheumatism, snake bite, stomach ache, ulcer of mouth, cholera, head ache, neuralgia and pain in legs |
| 9 | *Nerium oleander* L. | Apocynaceae | Leaves | S | Abortifacient, asthma, bronchitis, dysentery, gum trouble, gonorrhea, blisters, boils and eye disorder |
| 10 | *Calotropis gigantea* (L.) R.Br. | Asclepiadaceae | Latex, leaves, root, bark, juice, flower | S | Abortifacient, anasarca, anti-fertility, asthma, body ache, boils, burns, carbuncle, child, birth, dropsy, dysentery, ear complaints, epilepsy, eye complaints, guinea-worm, hydrophobia, injuries, intestinal worms, leprosy, purgative, rheumatism, ringworm, stomach ache, syphilis, tetanus, tooth ache, worms in gum and wounds |
| 11 | *Calotropis procera* (L.) W.T. Aiton | Asclepiadaceae | Root, bark, latex, flower, leaves, juice | S | Abortifacient, antidote in scorpion sting, antiseptic, asthma, cholera, cold, corn, cough, dropsy, eczema, epilepsy, fever, insect bite, laxative, leprosy, rheumatism, ringworm, scorpion bite, skin diseases, small pox, sores, spleen, complaints, stomach, diseases, swell, tooth ache, tooth worms, wounds, swell of mouth |
| 12 | *Cryptolepis buchananii* | Asclepiadaceae | Root, | S | Abdominal pain, anasarca, body ache, cholera, |

| | | | | | |
|---|---|---|---|---|---|
| | Roem. & Schult. | | leaves, stem, | | dropsy, dysentery, fits, internal fever, lactation, rheumatism, rickets in children, snake bite, stomach ache, syphilis and venereal diseases |
| 13 | *Ageratum conyzoides* L. | Asteraceae | Seed oil, leaves, juice, root | S | Anti-helmintic for stone, antiseptic, boils, burns, cuts, diarrhea, head ache, leprosy, muscular pain, piles, prolapsed anus, ringworm, scabies, skin diseases, snake bite, sores, body swelling, tumor, uterine disorder and lice in hairs |
| 14 | *Blumea lacera* DC. | Asteraceae | Leaves, juice, | H | Burns, cuts, wounds, bronchitis, fever, piles, toe injury, urine complaints, and fish poisoning |
| 15 | *Eclipta alba* Hassk. | Asteraceae | Whole plant, root, leaves | H | Antidote to snake bite, anti-fertility, antiseptic, asthma, bronchitis, cooling, eye disorder, fever, gastric, head ache, hepatic disorder, jaundice, leucoderma, liver complaints, malaria, promotes hair growth, scorpion sting, sores, spleen enlargement, tonic, tooth ache, ulcers and wounds |
| 16 | *Enydra fluctuans* Lour. | Asteraceae | Whole plant | H | Anasarca, blood pressure, dropsy and snake bite |
| 17 | *Sonchus arvensis* L. | Asteraceae | Whole plant | H | Cholera, cuts, wounds, dysentery and jaundice. |
| 18 | *Tridax procumbens* L. | Asteraceae | Whole plant | H | Antiseptic, blisters, boils, cuts & wounds, diarrhea, dysentery, eczema, eye disease, fever, leprosy, scorpion bite, skin diseases, sores, stomach ache, stone in bladder, tooth ache, ulcer and wounds |
| 19 | *Vernonia cinerea* Less. | Asteraceae | Whole plant | H | Cholera, dysentery, constipation, fever, impotency, lactation, malaria, night blindness, piles, skin diseases, threadworm, spleen complaints and wounds |
| 20 | *Xanthium strumarium* L. | Asteraceae | Root | S | Boils, wound, cooling, eye diseases, head ache, herpes, malaria, piles, rheumatism, ringworm, tooth ache, ulcer and urine complaints |
| 21 | *Carica papaya* L. | Caricaceae | Fruit, pulp of the leaves | SWT | Abortificient, palpitation bone fracture, constipation, cooling, digestive, gastric, ringworm, skin diseases, tooth & gum ache and urine bladder complaints |
| 22 | *Argyreia speciosa* (L.f.) Sweet. | Convolvulaceae | Root | S | Antidote, boils, diabetes, diarrhea, dysentery, gonorrhea, ringworm, head ache, small pox, sores, stomach complaints and syphilis |
| 23 | *Ipomoea aquatica* Forssk. | Convolvulaceae | Whole plant | H | Boils, eye disorder, gonorrhea, liver diseases, purgative and stomach disorder |
| 24 | *Ipomoea fistulosa* Mart. ex Choisy | Convolvulaceae | Root | S | Cuts, wounds, sprain and ulcer |
| 25 | *Ipomoea pes-tigrdis* L | Convolvulaceae | Root | H | Dog-bite, eye disease, fever, antidote to snake bite |
| 26 | *Bryonia laciniosa* L. | Cucurbitaceae | Seed | H | Asthma, carbuncle, cholera, colic, constipation, convulsion, eye infection, fever, head ache, complaints, promotes fertility in women, small pox, sores, spleen complaints, syphilis, tumor and snake bite |
| 27 | *Coccinia indica* Wight & Arn. | Cucurbitaceae | Root, fruit, leaves | H | Cold, convulsion, diabetes, filarial swelling, head ache, loss of taste, menorrhea, slow pulse, sores, syphilis and to stop vomiting |
| 28 | *Momordica charantia* L. | Cucurbitaceae | Root, fruit | CH | Anti-helmintic, cause vomiting, diabetes, eczema, laxative, malaria, edema, rheumatism and sores |
| 29 | *Momordica dioica* Roxb. ex Willd. | Cucurbitaceae | Root, fruit | CH | Anti-fertility, fever, injuries, pain in breast due to swelling, piles, asthma, bronchitis, delirium, dysentery, spleen complaints and head ache |
| 30 | *Phyllanthus niruri* L. | Euphorbiaceae | Root, | H | Allergy, boils, diarrhea, dropsy, dysentery, genito- |

| | | | | | |
|---|---|---|---|---|---|
| | | | whole plant | | urine disorder, indigestion, gastric disorder, jaundice, sores, swell and ulcers |
| 31 | *Abrus precatorius* L. | Fabaceae | Root, seed, leaves | C | Aphrodisiac, blood purifier, body pain, bronchitis, child birth, cold, cough, diarrhea, eczema, eye complaints, inflammations of gums, leucoderma, leucorrhoea, menstrual complaints, muscular pain, pneumonia, promotes conception, refrigerant, scabies,, scorpion bite, skin diseases, sore throat, stomach complaints, tonic, urine complaints, bone fracture and wounds |
| 32 | *Acacia arabica* (Lam.) Willd. | Fabaceae | Fruit, seed, bark, leaves | ST | Asthma, bronchitis, burns, cholera, diabetes, ear complaints, eye complaints, hair fall, head ache, leprosy, liver complaints, skin diseases, syphilis, throat infection and urine complaints |
| 33 | *Albizia lebbeck* (L.) Benth. | Fabaceae | Bark | T | Boils, carbuncle, diarrhea, dysentery, gonorrhea, eyesores, gum ulcer, night blindness and piles |
| 34 | *Bauhinia purpurea* L. | Fabaceae | Flower, seeds, bark | T | Astringent, bone fracture, indigestion, jaundice, small pox, tumor in stomach, wounds, anasarca, snake, tiger and scorpion bite, convulsion, dropsy, pain, poisoning by *Datura* and rheumatism |
| 35 | *Bauhinia variegata* L. | Fabaceae | Bark, root, leaves seeds | T | Diarrhea, dysentery, indigestion, laxative, leprosy, malaria, piles (bleeding), skin diseases, snake bite, syphilis, tumors, ulcers and worms |
| 36 | *Caesalpinia crista* L. | Fabaceae | Leaves, seed, root, bark | S | Colic fever, intermittent fever, malaria, menstrual complaints, pneumonia, skin diseases, swelling, tonic, pulmonary tuberculosis and wounds |
| 37 | *Cassia fistula* L. | Fabaceae | Fruit pulp, root, bark, flower, pods, leaves, root | MT | Abdominal pain, anti-fertility, antiseptic, asthma, blindness, blood purifier, burns, chest infection, constipation, cool agent, cough, diarrhea, dog bite, snake bite, dysentery, dysuria, epilepsy, indigestion, jaundice, leprosy complaints, pimples, rheumatism, ringworm, scorpion bite, skin diseases, stomach ache syphilis, tooth ache, vermicide and throat swelling |
| 38 | *Cassia occidentalis* L. | Fabaceae | Leaves, seeds, root | S | Bone fracture, diarrhea, dropsy, dysentery, eczema, fever, gastric complaints, purgative, rheumatism, ringworm, skin diseases, snake bite, throat infection, whooping cough and wounds. |
| 39 | *Cassia tora* L. | Fabaceae | Root, seeds, leaves | S | Abnormal child birth, anti-helmintic, antiseptic, boils, bone fracture, cold, eczema, epilepsy, fever, indigestion, intestinal disorder, itch, jaundice, itch, jaundice, night blindness, rheumatism, ringworm, scabies, scorpion bite, skin diseases, sores, stomach ache, vermicide and wounds |
| 40 | *Dalbergia sissoo* Roxb. | Fabaceae | Heart-wood, leaves | T | Antiseptic, dysentery, eruption, gonorrhea, headache, leprosy and skin diseases |
| 41 | *Desmodium gangeticum* DC. | Fabaceae | Whole plant | S | Antidote to snake venom, asthma, bronchitis, cough, dysentery, eye infection, fever and vomiting |
| 42 | *Desmodium triflorum* DC. | Fabaceae | Whole plant | H | Abscess, erupt, body ache, breast pain, colic, diarrhea, dysentery, menorrhea, sores, spleen complaints, nail disorder and tooth ache |
| 43 | *Mimosa pudica* L. | Fabaceae | Whole plant | S | Antidote to snake bite, anti-fertility, boils, sores, child birth, dysentery, epilepsy, fever, fistula, gum trouble, head ache, insomnia, gravel, leucorrhoea, loose motions, piles, scorpion bite, |

| | | | | | |
|---|---|---|---|---|---|
| | | | | | snake bite, tooth ache and wounds |
| 44 | *Mucuna prurita* Hook. | Fabaceae | Root, shoot, hairs | CH | Anasarca, anti-helmintic, aphrodisiac, appetizer, blisters, boils, ulcers, bone fracture, cough, dropsy, dysentery, eye disorder, impotency, night dreams, ringworm, roundworm, scorpion bite, snake bite, sore, syphilis, urine complaints and vermifuge |
| 45 | *Tamarindus indica* L. | Fabaceae | Fruit, leaves, seed | T | Anasarca, anti-helmintic, boils, cough, digestion, eye complaints, fever, head ache, muscular pain scorpion bite, stomach ache, sun stroke, swelling, syphilis, tooth ache and urine complaints |
| 46 | *Tephrosia purpurea* (L.) Pers. | Fabaceae | Whole, plant, root, seeds | S | Abortifacient, snake antidote, asthma, blood purifier, bronchitis, colic, dyspepsia, fever, gonorrhea, impotency, liver complaints, piles, spleen complaints, stomach ache, tonic, tooth ache, tumor and ulcer |
| 47 | *Abutilon indicum* (L.) Sweet. | Malvaceae | Root, seeds | S | Abortifacient, aphrodisiac, boil, bone fracture, bronchitis, child birth, colic, convulsion, cooling agent, cough, diarrhea, dysentery, gonorrhea, head ache, leprosy, paralysis, piles, purgative, stomach complaints, ulcer, arthritis and wounds |
| 48 | *Sida cordifolia* L. | Malvaceae | Root, seeds | S | Boils, dysentery, gonorrhea, leucorrhea, rheumatism, sexual weakness, stomachache, venereal disorder and wounds |
| 49 | *Urena lobata* L. | Malvaceae | Leaves, root, flower | S | Diarrhea, dysentery, hyperactivity, hydrophobia and lumbago |
| 50 | *Azadirachta indica* A. Juss | Meliaceae | Bark, root, young fruit, seeds, leaves, gum | T | Antipyretic, antiseptic, blood purifier, boils, cholera, diabetes, diarrhea, dysentery, dyspepsia, heart complaints, inflammation of gums, jaundice, leprosy, liver, complaints, malaria, measles, piles, rheumatism, scabies, skin disease, small pox, snake bite, spleen complaints, syphilis, tonic, tuberculosis, tumors, ulcer, urine complaints, venereal diseases and wounds |
| 51 | *Tinospora cordifolia* (Willd.) Miers. ex Hook. F. | Menispermacea e | Stem, root, leaves | S | Anti-periodic, antipyretic, aphrodisiac, asthma, bone fracture, cough, diarrhea, diphtheria, dysentery, fever, head ache, jaundice, malaria, piles, pulmonary tuberculosis, sex strength, skin diseases, tonic, venereal complaints and stomach diseases |
| 52 | *Moringa oleifera* Lamk. | Moringaceae | Root, bark, leaves, flower, fruit, seeds | T | Abortifacient, anti-fertility, appetizer, asthma, baldness, caries dental, catarrh, cholera, delivery, ear complaints, eczema, epilepsy, fever, head ache, intestinal worms, liver complaints, madness, menstrual complaints, paralysis, pneumonia, rheumatism, scabies, scorpion bite, scurvy, snake bite, sores, spleen enlargement, stomach ache, syphilis, tonic, typhoid, urine complaints and wounds |
| 53 | *Boerhavia diffusa* L. | Nyctaginaceae | Root | H | Abortifacient, anemia, asthma, blood purifier, body heat, child birth, cold, cough, dropsy, dysentery, eczema, epilepsy, eye, complaints, fever, fistula, gonorrhea, head ache, heart disorder, inflammation, jaundice complaints, liver complaints, menstrual complaints, pain in abdomen, piles, rheumatism, stomach complaints, urine complaints, and wounds |
| 54 | *Mirabilis jalapa* L. | Nyctaginaceae | Leaves, root | H | Blisters, boils, cold at child birth and piles |
| 55 | *Oxalis corniculata* L. | Oxalidaceae | Leaves | H | Cold, corn, cough, cuts, diarrhea, dysentery, epilepsy, eye complaints, fever, insect bite, |

| | | | | | |
|---|---|---|---|---|---|
| | | | | | jaundice, refrigerant, *Rickettsia*, scurvy, skin diseases and stomach ache |
| 56 | *Argemone mexicana* L. | Papaveraceae | Seed oil, leaves, juice, root | H | Boils, ulcer, arthritis, asthma, caries, cough, dog bite, dropsy, eye complaints,, gum trouble, head ache, indigestion, inflammation, jaundice, leprosy, mouthwash, piles, ringworm, scabies, skin diseases, syphilis, tooth ache and whooping cough |
| 57 | *Imperata cylindrica* Beauv. | Poaceae | Rhizome | H | Antidote to snake bite, fever, intestinal parasite, liver complaints, spleen complaints and piles |
| 58 | *Polygonum plebejum* R.Br. | Polygonaceae | Whole plant | H | Cough, diarrhea dysentery, gastric complaints, indigestion and lactation |
| 59 | *Portulaca oleracea* L. | Portulaceae | Whole plant | H | Blood purifier, burn, fever, head ache and burning sensation |
| 60 | *Portulaca quadrifida* L. | Portulaceae | Whole plant | H | Anti-scorbic, burns, ulcers, as cooling agent and skin diseases |
| 61 | *Anagallis arvensis* L. | Primulaceae | Juice | H | Cerebral, affection, hydrophobia and leprosy |
| 62 | *Ziziphus mauritiana* Lamk. | Rhamnaceae | Fruit, seed, kernel | S | Blood purifier, cholera, colic, diarrhea, digestion, dysentery, eye diseases (conjunctivitis), hair fall, fever, head ache, edema, rheumatism, scorpion sting, sores, spleen complaints, stomach ache, ulcer, wound and whooping cough |
| 63 | *Ziziphus nummularia* (Burm. f.) Wight. & Arn. | Rhamnaceae | Leaves, fruits | S | Boils, cold, diarrhea, dysentery, indigestion, inflammation of gums and tonic |
| 64 | *Aegle marmelos* Corr. | Rutaceae | Fruit, root, bark, stem, leaves, flower | T | Pain, abscess, astringent, back ache, mad dog bite, Breast pain, cholera, constipation, diabetes, diarrhea, dysentery, eye complaints, fever, gastric trouble, jaundice, laxative, nausea, night fever, palpitation, snake bite, stomach disorder, vomiting, wounds and cuts |
| 65 | *Murraya koenigii* (L.) Spreng. | Rutaceae | Root | S | Anti-helmintic, diarrhea, dysentery, eruption, laxative, malarial fever and stomach complaints |
| 66 | *Scoparia dulcis* L. | Scrophulariacea e | Leaves, whole plant | S | anti-emetic, digestive, eye diseases, fever, gonorrhea, child birth, rheumatism, sex weakness, tonic, ulcer of tongue and urine complaints |
| 67 | *Datura inoxia* Mill. | Solanaceae | Whole plant | S | Antihydrophobic, asthma, boils and rheumatism |
| 68 | *Datura metel* L. | Solanaceae | Whole plant, seeds, root, fruits | S | Anasarca, asthma, blisters, boils, bronchitis, cerebral complaints, cough, dropsy, epilepsy, fistula, gonorrhea, head ache, leprosy, madness, mums, piles, rheumatism, ringworm, skin diseases, small pox, snake bite, sores, syphilis, tumors, whooping cough and wounds |
| 69 | *Solanum nigrum* L. | Solanaceae | Whole plant | H | Antidote (opium), boils, cough, diarrhea, dysentery, ear complaints, eye complaints, fever, goiter, heart ailment, jaundice, liver complaints, inflammation of (scrotum, kidney and bladder), sores, nostril complaints, piles, rheumatism, skin diseases, sprain, stomach ache, swelling, throat trouble, ulcer in mouth and urine complaints |
| 70 | *Solanum surattense* Burm. f. | Solanaceae | Whole plant | H | Asthma, blisters, boils bronchitis, chest pain, cold, cough, dropsy, ear complaints, eye complaints, fever, gonorrhea, gum trouble, heart diseases, migraine, pain in jaw, paralysis, piles, scorpion bite, snake bite, sores, swelling, tooth ache and urine complaints |
| 71 | *Corchorus capsularis* L. | Tiliaceae | Leaves | H | Dysentery |
| 72 | *Phyla nodiflora* (L.) Greene | Verbenaceae | Fruit | H | Boils, diuretic, fever, menstrual complaints and fever |

C= Climber, S= shrub, H=Herb, St =Small tree, T= tree, SWT= Soft wooded tree, Mt= Medium tree, Ch=Climbing herb
